# Real-World Outcomes of Belinostat in Relapsed Refractory T-Cell Lymphomas: A Report from the Lymphoma Study Group of Turkish Society of Hematology

**DOI:** 10.3390/jcm15166350

**Published:** 2026-08-17

**Authors:** Meral Ulukoylu Menguc, Fatma Arikan, Pelin Aytan, Huseyin Saffet Bekoz, Fazıl Cagrı Hunutlu, Fatma Keklik Karadag, Gulcin Miyase Sonmez, Burak Deveci, Fatma Aykac, Ayse Uysal, Bugse Gulec Zengin, Turhan Koksal, Hulya Odluyurt, Deniz Goren, Turgay Ulas, Emel Isleyen, Esra Terzi Demirsoy, Metban Mastanzade, Selin Kucukyurt, Figen Atalay, Esin Oguz Kozan, Sureyya Yigit Kaya, Vildan Ozkocaman, Zehra Aksit Bozkına, Selami Koçak Toprak, Mustafa Cabuk, Volkan Karakus, Umut Yilmaz, Muhlis Cem Ar, Mehmet Sonmez, Melda Comert, Gulsum Ozet, Ozgur Mehtap, Mustafa Nuri Yenerel, Ahmet Kursad Gunes, Ayse Tulin Tuglular, Fahir Ozkalemkas, Güray Saydam, Isık Atagunduz, Tayfur Toptas

**Affiliations:** 1 Faculty of Medicine, Yeditepe University, 34718 Istanbul, Turkey; figen.atalay@yeditepe.edu.tr; 2 Faculty of Medicine, Marmara University, 34899 Istanbul, Turkey; fatmagecgelarikan@gmail.com (F.A.); ttuglular@gmail.com (A.T.T.); isikkaygusuz@yahoo.com (I.A.); toptast@gmail.com (T.T.); 3 Faculty of Medicine, Mersin University, 33110 Mersin, Turkey; drpelinaytan@gmail.com (P.A.); md.esinoguz@gmail.com (E.O.K.); 4 Medipol Mega University Hospital, Istanbul Medipol University, 34250 Istanbul, Turkey; drhsynbkz@gmail.com (H.S.B.); sureyyayigitkaya@hotmail.com (S.Y.K.); 5 Faculty of Medicine, Uludag University, 16059 Bursa, Turkey; fazilhunutlu@gmail.com (F.C.H.); vildanoz@uludag.edu.tr (V.O.); fahir@uludag.edu.tr (F.O.); 6 Faculty of Medicine, Ege University, 35100 Izmir, Turkey; fatma_keklik86@hotmail.com (F.K.K.); zehraaksit1993@hotmail.com (Z.A.B.); saydamguray@gmail.com (G.S.); 7 Faculty of Medicine, Ankara University, 06050 Ankara, Turkey; glcn_snmz@hotmail.com (G.M.S.); sktoprak@yahoo.com (S.K.T.); 8 Antalya Medstar Hospital, 07050 Antalya, Turkey; deveci.burak@gmail.com (B.D.); drmustafacabuk@gmail.com (M.C.); 9 Antalya Training and Research Hospital, 07100 Antalya, Turkey; fatmaaykas@hotmail.com (F.A.); dr_v_karakus@yahoo.com (V.K.); 10 Faculty of Medicine, Fırat University, 23200 Elazıg, Turkey; drayseorucuysal@gmail.com; 11 Cerrahpasa Faculty of Medicine, Istanbul University Cerrahpasa, 34098 Istanbul, Turkey; gulecbuse92@gmail.com (B.G.Z.); umutyza@hotmail.com (U.Y.); mcemar68@yahoo.com (M.C.A.); 12 Faculty of Medicine, Karadeniz Technical University, 61080 Trabzon, Turkey; dr.turhankoksal@hotmail.com (T.K.); mesonmez@yahoo.com (M.S.); 13 Gulhane Training and Research Hospital, 06010 Ankara, Turkey; hulyasazli@hotmail.com (H.O.); meldacomert@hotmail.com (M.C.); 14 Faculty of Medicine, Halic University, 34060 Istanbul, Turkey; drdenizgoren@gmail.com; 15 Abdurrahman Yurtaslan Oncology Hospital, 06200 Ankara, Turkey; turgayulas@yahoo.com; 16 Ankara Bilkent City Hospital, University of Health Sciences, 06800 Ankara, Turkey; dremel.isleyen@gmail.com (E.I.); gulsumozet@gmail.com (G.O.); 17 Faculty of Medicine, Kocaeli University, 41001 Kocaeli, Turkey; esraterzi@gmail.com (E.T.D.); ozgur.mehtap@thd.org.tr (O.M.); 18 Istanbul Faculty of Medicine, Istanbul University, 34093 Istanbul, Turkey; metban.mastanzade@istanbul.edu.tr (M.M.); mnyenerel@gmail.com (M.N.Y.); 19 Ankara Etlik City Hospital, University of Health Sciences, 06170 Ankara, Turkey; dr.skucukyurt@hotmail.com (S.K.); ahmetkgunes@gmail.com (A.K.G.)

**Keywords:** t-cell lymphoma, relapsed refractory, belinostat, HDAC inhibitors

## Abstract

**Background:** T-cell lymphomas (TCLs) constitute a heterogeneous group of mature T-cell neoplasms characterized by aggressive clinical behavior and poor outcomes, particularly in the relapsed/refractory (R/R) setting. Belinostat is a pan-HDAC inhibitor approved for R/R peripheral T-cell lymphomas. In this retrospective study, we analyze the real-world outcomes of single-agent belinostat in Turkish patients with R/R TCLs. **Methods:** Patients with R/R TCLs across Turkey who received at least one course of belinostat were included in the study. The primary end point was the overall response rate (ORR). The secondary end points included progression-free survival (PFS), overall survival (OS) and duration of response (DOR). **Results:** A total of 59 patients with R/R TCL were enrolled in the study, with a median age of 62 years. The median number of previous lines of therapy before belinostat was 2.5. The ORR for belinostat was 30.5% in the overall study cohort. Response assessment was available in 53 patients, among whom the ORR was 34% (18/53). The median PFS was 3 months. The median OS was 12 months, and the median DOR was not reached in responders. At least one adverse event occurred in 39.0% of patients. **Conclusions:** Belinostat produced objective responses in a subset of patients. These findings support belinostat as a potential individualized treatment option rather than a broadly effective standard for all patients with R/R TCLs. This multicenter real-world study demonstrates that the observed response rates closely mirror those reported in prospective clinical trials, confirming the reproducibility of belinostat efficacy in routine clinical practice.

## 1. Introduction

T-cell lymphomas (TCLs) comprise a rare group of non-Hodgkin lymphomas, and the outcomes are usually dismal compared to their B-cell counterparts [1,2]. Although new classifications were published in 2022, the classification of mature T-cell lymphomas did not undergo significant changes [3,4]. First-line therapy typically includes an anthracycline-based regimen featuring cyclophosphamide, doxorubicin, vincristine, and prednisone (CHOP). Unfortunately, this approach yields a low rate of complete responses, with a five-year survival rate of approximately 35% [5]. In young and fit patients, incorporating etoposide into the CHOP backbone has been shown to improve progression-free survival; however, this effect does not translate to a longer overall survival [6,7].

Although treatment options are evolving, the management of relapsed refractory (R/R) TCLs remains unsatisfactory. Current approaches include salvage chemotherapy followed by autologous or allogeneic stem cell transplantation (auto-SCT or allo-SCT), as well as single-agent novel therapies, followed by allo-SCT. Given the epigenetic dysregulation implicated in the pathogenesis of TCL, the role of histone deacetylase (HDAC) inhibitors as targeted therapies has become increasingly pronounced [8,9,10]. Belinostat is a pan-HDAC inhibitor approved for R/R peripheral T-cell lymphomas (PTCLs). Vorinostat and romidepsin are other approved HDAC inhibitors for TCLs. Belinostat monotherapy was approved for R/R PTCLs in 2017 based on the phase II BELIEF trial, demonstrating an overall response rate (ORR) of 25.8%, leading to a median progression-free survival (PFS) of 1.6 months and overall survival (OS) of 7.9 months [11].

The Global Peripheral T-cell Lymphoma consortium showed that single agents showed better survival outcomes compared to combination chemotherapy in the relapsed/refractory setting [12]. Although approved with the Phase 2 BELIEF trial, the real-world outcomes of belinostat for R/R TCLs have not been published. In this retrospective study, we analyze the real-world outcomes of single-agent belinostat in Turkish patients with R/R TCLs. The present multicenter retrospective study aimed to evaluate the real-world effectiveness and safety of belinostat in adult patients with R/R T-cell lymphoma and assess whether the outcomes reported in the pivotal BELIEF trial were reproducible in a heterogeneous routine-practice population.

## 2. Materials and Methods

Fifty-nine patients with RR TCLs from 19 centers across Turkey were analyzed retrospectively on behalf of the Lymphoma Study Group of the Turkish Society of Hematology. Clinical and follow-up data were obtained from medical records. Patients with R/R TCLs who received at least one course of belinostat at a dose of 1000 mg/m^2^ (administered on days 1–5 of 21-day cycles) were included in the study. Patients who received belinostat for less than one course or those who received belinostat in combination with chemotherapy were excluded. Patient demographics, disease characteristics, response to belinostat, and adverse event profiles were analyzed.

The primary end point was the ORR. The secondary end points included PFS, OS and duration of response (DOR). Response to belinostat was assessed by treating investigators using available clinical and radiological data, including PET/CT according to the 2014 Lugano Response Criteria for NHLs [13]. The timing of assessment was based on local clinical practice, generally after 2 cycles or at approximately 12 weeks, and earlier when clinically indicated. Because of the retrospective multicenter design, no central radiological or pathological review was performed. The ORR was calculated by adding the complete response (CR) rate to the partial response (PR) rate. The DOR was defined as the time from the first documented objective response (complete or partial response) to disease progression or death, whichever occurs first. PFS was calculated from the date of belinostat initiation to the date of documented disease relapse or progression, or the date of death, whichever occurred first. OS was calculated from the date of belinostat initiation to the date of death from any cause; for surviving patients, the data were censored at their last known alive visit. Adverse events (AEs) were defined as clinical or laboratory events recorded during belinostat treatment. Treatment-emergent adverse events (TEAEs) were those considered by the treating investigator to be related or possibly related to belinostat; toxicity-related deaths were defined separately as deaths attributed by the participating center to treatment-related complications. AEs and TEAEs were analyzed from medical records and graded according to the Common Terminology Criteria for Adverse Events (CTCAE) version 5.0. The research protocol was approved by the local ethics committee.

During the preparation of this manuscript, the authors used ChatGPT-5.6 for English editing. The authors have reviewed and edited the output and take full responsibility for the content of this publication.

### Statistical Methods

Statistical analyses were performed using “IBM SPSS Statistics for Windows. Version 25.0 (Statistical Package for the Social Sciences, IBM Corp., Armonk, NY, USA)” [14]. Descriptive statistics are presented as *n* and % for categorical variables and median (min-max) for continuous variables. The Kaplan–Meier method was used to calculate overall survival and PFS durations. Univariate and multivariate Cox regression analysis results on the risk of death and progression of various variables are presented. Variables reaching statistical significance in univariable analyses (*p* < 0.05) were included in the multivariable Cox regression model. No imputation of missing data was performed.

## 3. Results

A total of 36 male and 23 female relapsed refractory patients with T-cell lymphoma treated at 19 centers in Turkey were enrolled in the study, with a median age of 62 years (range: 23–80 years). The most frequent types of TCLs included peripheral T-cell lymphoma, not otherwise specified (PTCL, NOS), and angioimmunoblastic T-cell lymphoma (AITL). Anaplastic large cell lymphoma and transformed mycosis fungoides were among the remaining types. The vast majority of patients were in an advanced stage, with Ann-Arbor stage IV disease detected in 61.0%, followed by stage III (23.7%). When prognostic risk scores were examined, according to the IPI scoring, more than half of the patients were in the high/moderate risk group (50.8%). According to the INT T-cell project score, 43.1% of the patients were assessed as moderate risk and 15.5% as high risk. The demographics of the patients are summarized in Table S1.

Anthracycline-containing chemotherapy, with or without etoposide, was the most preferred first-line therapy. Most patients received salvage chemotherapy containing Brentuximab vedotin as the second line of treatment (62.2%). The median number of previous lines of therapy before belinostat was 2.5 (range: 1–9), and the median number of belinostat cycles was 2 (range: 1–58). Only 14 patients (24%) received prior auto-SCT and 2 patients received prior allo-SCT. Eight patients (13%) proceeded to allogeneic stem cell transplantation (allo-SCT) after belinostat.

Response assessment was unavailable in six patients (10.2%), mainly because of early treatment discontinuation before the first formal response evaluation. Accordingly, the ORR was 30.5% (18/59) in the overall cohort and 34.0% (18/53) among response-evaluable patients (Table S2a,b). At the time of data analysis, 46% of patients were alive. The cause of death was disease progression in 71% of cases, while 9.7% died due to reasons other than lymphoma progression or toxicities. Deaths related to toxicity occurred in six patients (19.4%) (Table S2a).

Regarding belinostat-related toxicity, at least one adverse event occurred in 39.0% of patients. The most frequent toxicity was neutropenia (36.4%), followed by transaminitis (18.2%) and lymphopenia (13.6%). When toxicity levels were examined, grade 2 adverse events were most common (40.9%); grade 3–5 toxicity accounted for a total of 45.4%; and grade 5 toxicity was reported in one patient (4.5%) (Table S2).

The median follow-up period was 9 months (range: 1–84 months). Table S3 presents the Kaplan–Meier survival analysis results, which evaluate the OS and PFS outcomes of the 59 patients with RR TCLs included in the study. The median PFS was 3 months (95% CI: 2.0 to 6.0). PFS at 2 years was reported as 22.5%. Five-year progression-free survival rates have not been reported at a calculable level; this may be related to the high number of events and the limited number of patients followed up long-term without progression. The median OS was 12 months (95% CI: 8 to 22). When survival probabilities were examined, the 2-year overall survival rate was found to be 33.7%, and the fact that the 5-year survival rate also remained at 33.7%. The median duration of response (mDOR) was not reached in responders (patients having a response of ≥PR to belinostat) (Figure S1).

Tables S4 and S5 show the effects of various clinical variables on survival (OS and PFS respectively) evaluated using univariate and multivariate Cox regression analyses. Univariate analyses examined numerous prognostic variables related to the patient, disease, and treatment. In multivariate analysis, neither OS nor PFS showed correlation with gender, Ann Arbor stage, IPI, prior auto-SCT, proceeding to allo-SCT, or the number of previous lines of therapy (Tables S4 and S5).

In the multivariable Cox regression model, objective response (CR/PR), treatment-related adverse events, and receiving more than two cycles of belinostat remained statistically associated with overall survival. Patients achieving an objective response had a significantly lower risk of death (HR = 0.273, 95% CI 0.079–0.947; *p* = 0.041), whereas patients receiving ≤2 cycles of belinostat had a higher mortality risk (HR = 3.554, 95% CI 1.314–9.610; *p* = 0.012). Likewise, the occurrence of treatment-related adverse events was associated with inferior survival (HR = 4.038, 95% CI 1.887–8.643; *p* < 0.001).

In the multivariable model for progression-free survival, objective response and receiving more than two cycles of belinostat remained significantly associated with longer PFS. Patients achieving CR/PR had a markedly lower risk of progression (HR = 0.092, 95% CI 0.021–0.416; *p* = 0.002), whereas treatment limited to ≤2 cycles was associated with a significantly increased progression risk (HR = 4.387, 95% CI 1.635–11.771; *p* = 0.003).

## 4. Discussion

TCLs constitute a biologically heterogeneous group of mature T-cell neoplasms characterized by aggressive clinical behavior and poor outcomes, particularly in the relapsed/refractory (R/R) setting. Despite recent therapeutic advances, treatment options are limited after failure of anthracycline-based chemotherapy, and survival following relapse is generally dismal. The standard of care for RR TCLs is lacking because the rarity and aggressiveness of these lymphomas make it difficult to obtain strong evidence. Most of the data comes from observational cohorts or early-phase trials. Belinostat is a pan-HDAC inhibitor approved for RR peripheral T-cell lymphomas and has been available in Turkey since 2019. In this multicenter real-world study, we evaluated the efficacy and safety of belinostat in 59 patients with RR TCL treated across 19 centers in Turkey. To our knowledge, this study represents the first dedicated real-world analysis of belinostat in RR TCLs and one of the largest multicenter datasets reported outside prospective clinical trials. To date, only an online poster abstract from Brazil was reported in 2025, but the manuscript has not been published yet [15]. Our findings demonstrate that belinostat retains clinically meaningful activity in routine clinical practice, with an ORR of 30.5%, median PFS of 3 months, and median OS of 12 months. These results provide important external validation of the efficacy observed in prospective studies while also highlighting the challenges that remain in this high-risk population [11,16].

The prevalence of specific T-cell lymphoma subtypes, particularly peripheral T-cell lymphoma, not otherwise specified (PTCL and NOS), and angioimmunoblastic T-cell lymphoma (AITL), was similar to previous reports [11,15], highlighting the heterogeneity within T-cell neoplasms and reinforcing the importance of accurate diagnosis and tailored therapeutic strategies.

The distribution of patients with advanced-stage disease was similar to the phase 2 BELIEF cohort [11]. This is critical, as advanced-stage presentations typically correlate with poorer prognoses and necessitate more aggressive treatment approaches [16,17]. In terms of prognostic assessment, the IPI scores revealed that over half of our cohort had a score of 3, which further substantiates the challenges encountered in this patient population.

The use of anthracycline-based chemotherapy as the primary treatment option and subsequent utilization of Brentuximab vedotin in salvage therapy reflects current clinical practices [7,11,16,17,18]. Notably, the median number of prior treatment lines in our cohort was 2.5, comparable to that reported in BELIEF, suggesting that the observed efficacy is not simply attributable to treatment in earlier disease stages [19].

Given the retrospective nature of the study, no formal a priori sample size calculation was performed. With 59 patients, the observed ORR was 30.5% 95% CI: 19.2–43.9%, corresponding to 18 responders and indicating its potential efficacy in managing relapsed refractory T-cell lymphoma, although the response rate can be considered modest when contrasted with therapies used in other lymphomas [16,17,20]. Assuming a historical ORR of 25.8 regarding the BELIEF trial, the estimated post hoc power to detect this difference was low, approximately 13% using a two-sided alpha of 0.05. The median PFS of 3 months and median OS of 12 months illustrate the aggressive nature of this malignancy, coupled with the emerging need for refining treatment approaches to enhance both survival and quality of life. Median PFS and OS were longer than in the BELIEF trial in our cohort, which could be attributed to the number of patients with highly aggressive TCL types such as enteropathy-associated TCL or hepatosplenic TCL in the original cohort. Our cohort did not include these aggressive types of TCL, whereas 10% of our patients consisted of transformed mycosis fungoides. Another noteworthy aspect of our findings relates to the biological heterogeneity of TCLs and the potential role of epigenetic vulnerability in determining sensitivity to HDAC inhibition. In recent years, substantial evidence has demonstrated that AITL and other T-follicular helper (TFH)-derived lymphomas are characterized by recurrent mutations involving epigenetic regulators, including TET-2, DNMT3A, IDH2 and RHOA. These alterations result in widespread epigenetic dysregulation and provide a strong biological rationale for the use of epigenetic therapies such as HDAC inhibitors and hypomethylating agents. Another possible explanation for numerically longer PFS and OS besides ORR compared to the BELIEF cohort could be the relatively higher proportion of patients with AITL in our cohort, which has previously been shown to be well responding to HDACs [21,22,23,24,25]. Our cohort included 17 (28.8%) patients with AITL, whereas 22 (18.3%) patients with AITL were enrolled in the original study.

Interestingly, while several factors including gender, Ann Arbor stage, IPI, number of previous lines of therapy, and prior stem cell transplantation status were analyzed, no significant correlations were identified with PFS or OS. The 2-year overall survival rate was found to be 33.7%, and the fact that the 5-year survival rate also remained at 33.7%. Because of the limited follow-up duration and the small number of patients remaining at risk beyond two years, these estimates should be interpreted cautiously.

An important methodological limitation of the present study is that objective response and the number of belinostat cycles were analyzed as fixed covariates in conventional Cox regression models despite being treatment-dependent variables. Patients with early progression or death inevitably receive fewer treatment cycles and may not survive long enough to undergo formal response assessment. Consequently, the observed associations between longer treatment duration, objective response, and improved survival may partly reflect reverse causality, guarantee-time bias, and immortal-time bias rather than true independent prognostic effects. Time-dependent Cox regression or landmark analyses would provide a more robust assessment of these variables but were not feasible because of the retrospective design and limited sample size. Therefore, these findings should be considered exploratory and interpreted with appropriate caution.

Because both objective response and treatment duration are post-baseline variables that become observable during follow-up, these findings should be interpreted as exploratory associations rather than evidence of independent causal prognostic effects. Patients with early progression or death are inherently less likely to receive multiple treatment cycles or undergo response assessment, which may introduce guarantee-time and immortal-time bias. Therefore, these variables primarily reflect the clinical course after treatment initiation rather than baseline prognostic characteristics.

However, these variables are treatment-dependent and occur after treatment initiation. Consequently, the observed associations may partially reflect reverse causality, guarantee-time bias, and immortal-time bias, since patients experiencing early progression are less likely to receive additional treatment cycles or achieve an objective response. These findings should therefore be interpreted as hypothesis-generating rather than definitive evidence of independent prognostic effects.

Although the evidence for the benefit of allo-SCT in RR TCLs is limited, recent data reports that proceeding to allo-SCT shows higher survival rates than novel agents or salvage chemotherapy [26,27,28]. An important and potentially controversial finding of our analysis is the lack of a statistically significant survival advantage among patients proceeding to allo-SCT. Eight patients underwent allo-SCT following belinostat treatment, yet no significant differences in PFS or OS were observed compared with non-transplanted patients. Several considerations are essential when interpreting this result. First, the number of transplanted patients was small, substantially limiting statistical power and increasing the likelihood of a type II error. Second, allo-SCT recipients often represent a highly selected population characterized by either particularly favorable treatment responses or, conversely, more aggressive disease requiring consolidation. Such competing selection mechanisms may obscure true transplant effects in retrospective analyses. Third, immortal-time bias represents a major methodological concern. By definition, patients must survive long enough to undergo transplantation, creating a time-dependent exposure that cannot be fully accounted for in conventional Cox regression models unless transplantation is treated as a time-varying covariate. Finally, heterogeneity in conditioning regimens, donor sources, and timing of transplantation may further dilute measurable effects. Therefore, our findings should not be interpreted as evidence against the utility of allo-SCT but rather as an indication that larger datasets with appropriate time-dependent statistical modeling are required to define the role of transplantation following belinostat-induced responses.

The safety profile observed in our cohort was generally consistent with previous reports [11,29]. Hematologic toxicities, particularly neutropenia, represented the most common adverse events, while hepatic enzyme elevations and lymphopenia occurred less frequently. Most recorded TEAEs were clinically manageable; however, six deaths were considered treatment-related by the treating investigators, predominantly due to severe infectious complications. Given the retrospective multicenter design and the incomplete capture of fatal adverse events in the safety dataset, these findings should be interpreted with caution. Nevertheless, they underscore the importance of vigilant monitoring for infectious complications and prompt supportive care during belinostat treatment.

The present study possesses several notable strengths. First, it reflects routine clinical practice across multiple institutions, thereby enhancing external validity. Second, the inclusion of heavily pretreated patients provides insight into a population frequently underrepresented in prospective studies. Third, the multicenter design reduces the influence of institution-specific treatment practices. Most importantly, this study addresses a significant gap in the literature by providing one of the first real-world assessments of belinostat in R/R TCL. While prospective trials establish efficacy under controlled conditions, real-world studies are essential for understanding treatment performance in broader patient populations and for informing everyday clinical decision-making.

Several limitations should also be acknowledged. The retrospective design inherently introduces risks of selection bias, information bias, and residual confounding. Central pathology review was not performed, potentially resulting in diagnostic heterogeneity. Response assessments were conducted according to local institutional practice rather than centralized radiologic review. Molecular profiling data were unavailable for most patients, preventing evaluation of genomic predictors of response. Finally, the relatively modest sample size limited statistical power for subgroup analyses, particularly regarding histologic subtype-specific outcomes and transplantation effects. These limitations should be considered when interpreting our findings.

In conclusion, this multicenter real-world study provides clinically relevant evidence regarding belinostat use in heavily pretreated patients with R/R TCL. Belinostat produced objective responses in a subset of patients, but disease control was generally limited and substantial heterogeneity was observed across the cohort. These findings support belinostat as a potential individualized treatment option rather than a broadly effective standard for all patients with R/R TCLs. Its most meaningful clinical role may be in selected patients who are unsuitable for intensive salvage approaches, require a non-cytotoxic treatment option, or may benefit from temporary disease control as a bridge to further therapy. Prospective studies incorporating centralized pathology review, standardized response assessment, biospecimen collection, and molecular profiling are required to identify predictive biomarkers and define the subgroups most likely to derive durable benefit. Although no survival advantage associated with allo-SCT was observed, methodological limitations and limited statistical power preclude definitive conclusions regarding transplant benefit. The biological susceptibility of AITL and TFH-derived lymphoma to epigenetic therapies further supports the continued development of HDAC inhibitor-based approaches [30].

As one of the first real-world studies of belinostat in TCL, our findings provide valuable evidence supporting its role in contemporary practice and underscore the need for prospective biomarker-driven studies aimed at optimizing patient selection and improving outcomes in this challenging disease.

## Data Availability

The raw data supporting the conclusions of this article will be made available by the authors upon request.

## Supplementary Materials

The following supporting information can be downloaded at: https://www.mdpi.com/article/10.3390/jcm15166350/s1, Figure S1: Kaplan–Meier graphics for DOR, PFS and OS for total cohort; Figure S2: Overall Survival by response status; Figure S3: PFS by response status Table S1: Characteristics of patients; Table S2a: Summary of belinostat efficacy and toxicity in evaluable patients; Table S2b: Summary of belinostat efficacy according to histological type; Table S3: Overall survival and progression-free survival for total cohort; Table S4: Multivariate analysis for overall survival; Table S5: Multivariate analysis for progression-free survival.
