# Supplementary material for: Real-World Outcomes of Belinostat in Relapsed Refractory T-Cell Lymphomas: A Report from the Lymphoma Study Group of Turkish Society of Hematology
Source: J Clin Med. 2026 Aug 17;15(16):6350. doi: 10.3390/jcm15166350 (PMC13513793; doi:10.3390/jcm15166350)
Supplement: Supplementary file 1 [file jcm-15-06350-s001.zip › SUPPLEMENTART BELINOSTAT/TABLES belinostat(1).pdf]

Characteristic	N = 59
**Gender**	
Male	36 (61%)
Female	23 (39%)
**Disease Type**	
PTCL,NOS	20 (34%)
AITL	17 (29%)
ALCL	6 (10%)
Transformed MF	6 (10%)
Other	10 (17%)
**Ann-Arbor stage**	
1	3 (5.1%)
2	6 (10%)
3	14 (24%)
4	36 (61%)
**ECOG score**	
0	19 (32%)
1	25 (42%)
2	14 (24%)
3	1 (1.7%)
**LDH**	
Normal	14 (24%)
Elevated	44 (75%)
Unknown	1 (1.7%)
**IPI Score**	
1	14 (24%)
2	10 (17%)
3	30 (51%)
4	5 (8.5%)
**Int_T cell_score**	
1	24 (41%)
2	25 (43%)
3	9 (16%)
Unknown	1
**First line treatment**	
CHOEP	16 (31%)
CHOP	25 (49%)
BV-CHP	9 (18%)
Other	1 (2.0%)
Unknown	8
**Second line treatment**	
BV-based	23 (62%)
ICE /DHAP	2 (5.4%)
Gem-based	4 (11%)
Other	8 (22%)
Unknown	22
**Previous ASCT**	
No	45 (76%)
Yes	14 (24%)
**Allo-SCT**	
No	48 (83%)
Yes	10 (17%)
Unknown	

Table S1: Characteristics of patients (Abbreviations : PTCL ,NOS ; Peripheral T-Cell Lymphoma , not otherwise specified , AITL ; Angioimmunoblastic T-Cell Lymphoma ,ALCL ; Anaplastic large-cell lymphoma , MF ; Mycosis Fungoides , IHC: immunohistochemical staining , ECOG ; Eastern Cooperative Oncology Group Performance Status , LDH ; Lactate dehydrogenase , IPI ; International Prognostic Index , Int T cell score ; International T cell score , CHOEP ; cyclophosphamide ,doxorubicine , vincristine ,etoposide and prednisolone , CHOP ; cyclophosphamide ,doxorubicine , vincristine and prednisolone , BV-CHP ; Brentuximab vedotin, cyclophosphamide ,doxorubicine and prednisolone , BV ; Brentuximab vedotin, ICE ; Ifosfamide ,carboplatine etoposide , DHAP ; Cisplatin ,cytarabine ,dexamethasone , Gem; Gemcitabine , ASCT : Autologous stem cell transplantation , allo-SCT : Allogeneic stem cell transplantation

Characteristic	N = 59
**Response to belinostat**	
Unknown	6 (10.2 %)
CR	7 (11.9 %)
PR	11 (18.6 %)
SD	6 (10.2 %)
PD	29 (49.2 %)
**TEAE**	
Absent	36 (61%)
Present	23 (39%)
**TEAE Type**	
Nausea	1 (4.5%)
Phlebitis	1 (4.5%)
Cardiac failure	1 (4.5%)
Lymphopenia	3 (13.6 %)
Mucositis	1 (4.5%)
Neutropenia	8 (36.4 %)
Pneumonia	1 (4.5%)
Sepsis	2 (9.1%)
Transaminitis	4 (18.2%)
**Side_effect_grade**	
1	3 (13.6%)
2	9 (40.9 %)
3	7 (31.8 %)
4	2 (9.1%)
5	1 (4.5%)
**Progression**	
No	16 (27%)
Yes	43 (73%)
**Mortality**	
Live	27 (46%)
Ex	32 (54%)
**Cause of death**	
Disease progression	22 (71%)
Toxicity	6 (19%)
Other than lymphoma	3 (9.7%)

Table S2a : Summary of Belinostat efficacy and toxicity in evaluable patients (Abbreviations : CR; Complete response , PR ; Partial response , SD ; Stabile disease , PD ; Progressive disease , TEAE : Treatment emergent adverse event)

DISEASE SUBTYPE	n	CR	PR	ORR (%)

PTCL,NOS	20	1	5	30
AITL	17	3	2	29
ALCL	6	2	0	33
Transformed MF	6	0	1	16
Other	10	1	4	50

Table S2 b: Summary of Belinostat efficacy according to histological type (Abbreviations : PTCL ,NOS ; Peripheral T-Cell Lymphoma , not otherwise specified , AITL ; Angioimmunoblastic T-Cell Lymphoma ,ALCL ; Anaplastic large-cell lymphoma , MF ; Mycosis Fungoides

**Because of the limited number of patients within each histological subtype , these results are presented desciptively without formal statistical comparisons

Endpoint	N	Event	Censored	Median months (%95 CI)	2 years %	5 years %
OS	59	32	27	12.00 (8.00-22.00)	33.7	33.7
PFS	59	43	16	3.00 (2.00-6.00)	22.8	—

Table S3: Overall survival and progression free survival for total cohort

Parameter	Group	Univariate HR (%95 CI)	p	Multivariate HR (%95 CI)	p
**Gender**	Female	Ref.		—	—
	Male	1.680 (0.776-3.638)	0.188	—	—
**Ann Arbor stage**	Stage I	Ref.		—	—
	Stage II	0.396 (0.066-2.385)	0.312		
	Stage III	0.953 (0.250-3.630)	0.944	—	—
	Stage IV	0.691 (0.203-2.350)	0.554	—	—
**Ann Arbor early / advanced stage**	Stages I-II	Ref.		—	—
	Stages III-IV	1.213 (0.466-3.154)	0.693	—	—
**IPI score**	Low	Ref.		—	—
	Low - intermediate	1.621 (0.542-4.849)	0.387	—	—
	High - intermediate	1.439 (0.596-3.476)	0.418	—	—
	High	1.087 (0.225-5.250)	0.917	—	—
**INT-T cell project score**	Low	Ref.		—	—
	Intermediate	1.393 (0.635-3.058)	0.408	—	—
	High	1.399 (0.477-4.102)	0.541	—	—
**CD30 IHC staining**	Negative	Ref.		—	—
	Positive	1.123 (0.504-2.504)	0.777	—	—
**Auto-SCT**	Yes	Ref.		—	—
	No	1.518 (0.718-3.210)	0.275	—	—
**Allo-SCT**	No	Ref.		—	—
	Yes	0.480 (0.168-1.373)	0.171	—	—
**Previous lines of therapy**	Per 1 line	0.919 (0.717-1.177)	0.502	—	—
**Number of Belinostat cycles**	Per 1 number	0.749 (0.594-0.944)	**0.015**	—	—
**Response status to Belinostat**	Non-responder	Ref.		—	—
	(CR/PR)	0.183 (0.064-0.526)	**0.002**	0.273 (0.079-0.947)	**0.041**
**TEAE with Belinostat**	Absent	Ref.		—	—
	Present	2.182 (1.086-4.384)	**0.028**	4.038 (1.887-8.643)	**<0.001**
**Disease Type**	PTCL-NOS	Ref.		—	—
	AITL	0.680 (0.261-1.769)	0.429	—	—
	ALCL	0.836 (0.233-3.003)	0.783		

	MF	1.564 (0.497-4.921)	0.445	—	—
	Other	1.092 (0.422-2.822)	0.857	—	—
**1st line therapy**	CHOEP	Ref.		—	—
	CHOP	1.429 (0.603-3.389)	0.418	—	—
	BV-CHP	0.889 (0.267-2.956)	0.848	—	—
**Number of Belinostat cycles**	>2.00	Ref.		—	—
	≤2.00	4.521 (1.998-10.230)	**<0.001**	3.554 (1.314-9.610)	**0.012**
				**-2 Log Likelihood=198.70, p<0.001**	

Table S4: Multivariate analysis for overall survival (Abbreviations : IPI; International prognostic index , Int. T-cell ; International T-cell project , Auto-SCT : Autologous stem cell transplantation , Allo-SCT ; Allogeneic stem cell transplantation, IHC : immunohistochemical staining , TEAE; Treatment Emergent Adverse Event , PTCL ,NOS ; Peripheral T-Cell Lymphoma , not otherwise specified , AITL ; Angioimmunoblastic T-Cell Lymphoma ,ALCL ; Anaplastic large-cell lymphoma , MF ; Mycosis Fungoides , CHOEP ; cyclophosphamide ,doxorubicine , vincristine ,etoposide and prednisolone , CHOP ; cyclophosphamide ,doxorubicine , vincristine and prednisolone , BV-CHP ; Brentuximab vedotin, cyclophosphamide ,doxorubicine and prednisolone

**Variable**	**Group**	**Univariate HR (%95 CI)**	**p**	**Multivariate HR (%95 CI)**	**p**
**Sex**	Female	Ref.		—	—
	Male	0.886 (0.479-1.642)	0.701	—	—
**Ann Arbor stage**	Stage I	Ref.		—	—
	Stage II	0.200 (0.033-1.221)	0.081		
	Stage III	0.813 (0.225-2.934)	0.752	—	—
	Stage IV	0.576 (0.171-1.943)	0.374	—	—
**Ann Arbor Advanced Stage**	Stage I-II	Ref.		—	—
	Stage III-IV	1.637 (0.643-4.169)	0.302	—	—
**IPI score**	Low	Ref.		—	—
	Low - intermediate	0.995 (0.376-2.629)	0.992	—	—
	High - intermediate	0.918 (0.431-1.952)	0.823	—	—
	High	1.504 (0.471-4.798)	0.491	—	—
**INT-T cell project score**	Low	Ref.		—	—
	Intermediate	0.950 (0.489-1.845)	0.879	—	—
	High	1.155 (0.478-2.794)	0.749	—	—
**Auto-SCT**	No	Ref.		—	—
	Yes	1.122 (0.563-2.236)	0.743	—	—
**Allo-SCT**	No	Ref.		—	—
	Yes	0.426 (0.167-1.089)	0.075	—	—
**Number of lines prior to Belinostat**	Per 1 line	0.899 (0.740-1.092)	0.282	—	—
**Number of Belinostat cycles**	Per 1 cycle	0.731 (0.603-0.885)	**0.001**	—	—
**Response to Belinostat**	Non-responder	Ref.		—	—
	Responder (CR/PR)	0.096 (0.037-0.254)	**<0.001**	0.092 (0.021-0.416)	**0.002**
**TEAE with Belinostat**	Absent	Ref.		—	—
	Present	1.236 (0.666-2.294)	0.502	—	—
**Disease Type**	PTCL-NOS	Ref.		—	—
	AITL	0.757 (0.348-1.647)	0.483	—	—
	ALCL	0.827 (0.275-2.484)	0.735		
	MF	0.827 (0.275-2.484)	0.735	—	—
	Other	0.913 (0.375-2.224)	0.841	—	—

**1st line treatment**	CHOEP	Ref.		—	—
	CHOP	1.149 (0.546-2.415)	0.715	—	—
	BV-CHP	1.035 (0.381-2.815)	0.946	—	—
**Number of Belinostat cycles**	>2.00	Ref.		—	—
	≤2.00	8.288 (3.916-17.540)	**<0.001**	4.387 (1.635-11.771)	**0.003**
				**-2 Log Likelihood=128.27, p<0.001**	

Table S5: Multivariate analysis for progression free survival (Abbreviations : IPI; International prognostic index , Int. T-cell ; International T-cell project , Auto-SCT : Autologous stem cell transplantation , Allo-SCT ; Allogeneic stem cell transplantation, IHC : immunohistochemical staining , TEAE; Treatment Emergent Adverse Event , PTCL ,NOS ; Peripheral T-Cell Lymphoma , not otherwise specified , AITL ; Angioimmunoblastic T-Cell Lymphoma ,ALCL ; Anaplastic large-cell lymphoma , MF ; Mycosis Fungoides , CHOEP ; cyclophosphamide ,doxorubicine , vincristine ,etoposide and prednisolone , CHOP ; cyclophosphamide ,doxorubicine , vincristine and prednisolone , BV-CHP ; Brentuximab vedotin, cyclophosphamide ,doxorubicine and prednisolone
