# Supplementary material for: Real-World Outcomes of Belinostat in Relapsed Refractory T-Cell Lymphomas: A Report from the Lymphoma Study Group of Turkish Society of Hematology
Source: J Clin Med. 2026 Aug 17;15(16):6350. doi: 10.3390/jcm15166350 (PMC13513793; doi:10.3390/jcm15166350)
Supplement: Supplementary file 1 [file jcm-15-06350-s001.zip › SUPPLEMENTART BELINOSTAT/Figures Belinostat.pdf]

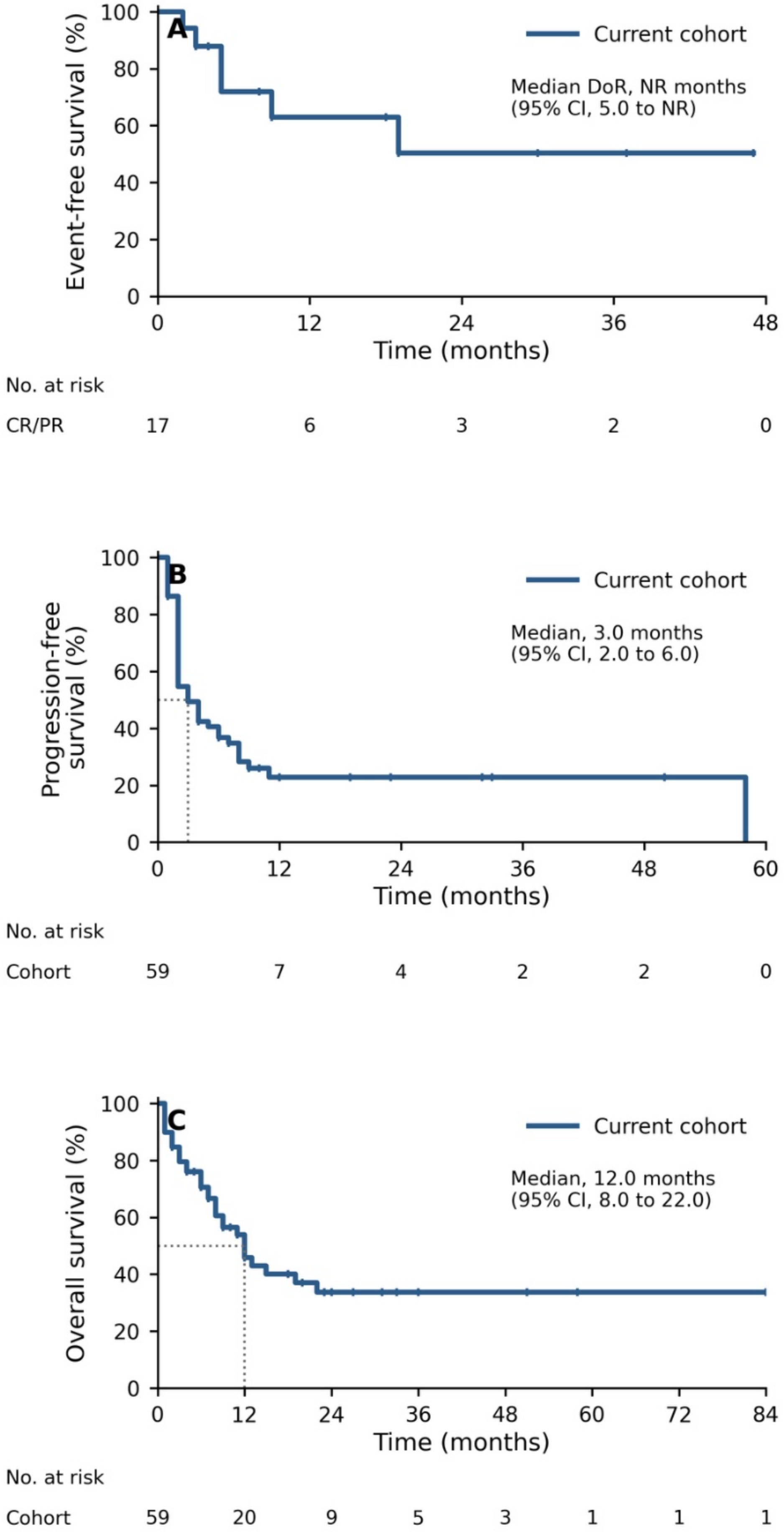

Scanned with CamScanner

Figure S1: Kaplan-Meier graphics for DOR ,PFS and OS for total cohort

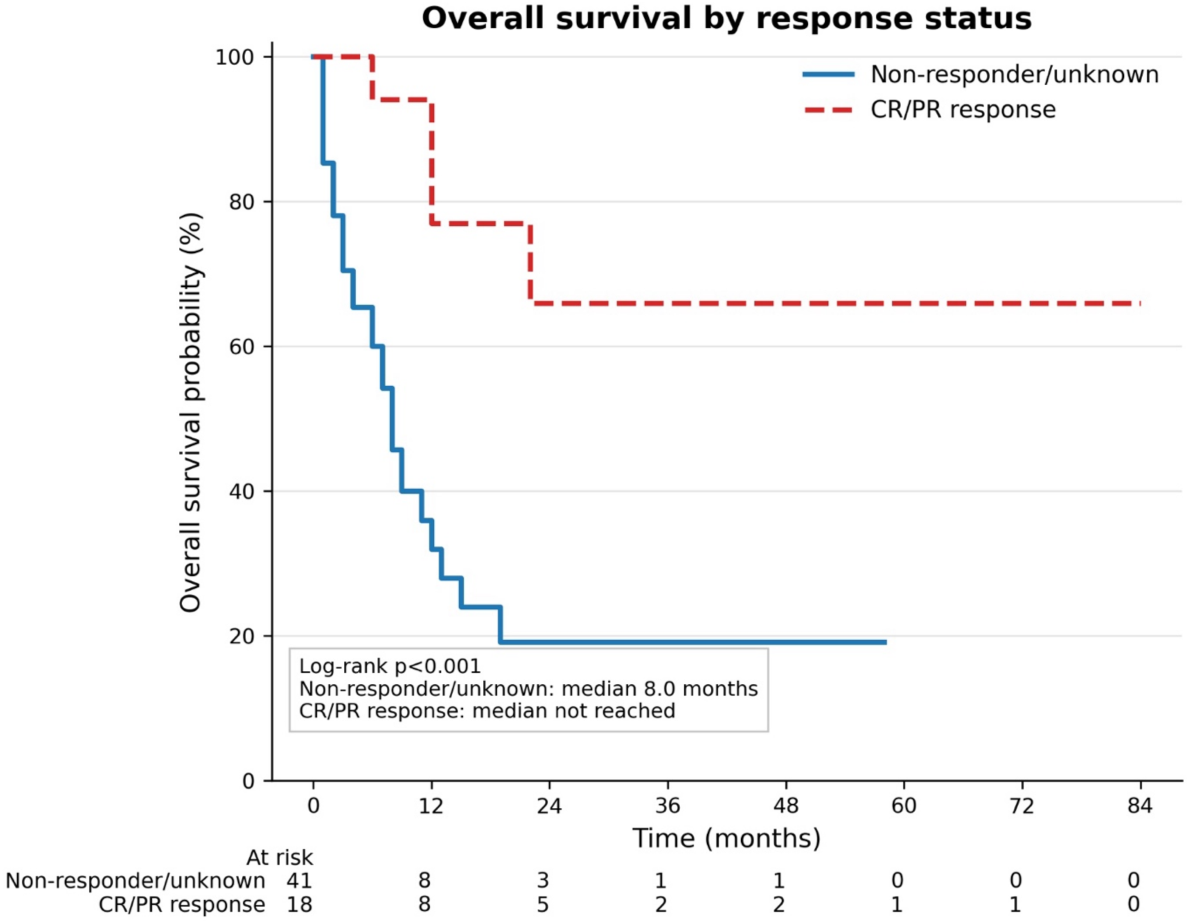

Figure S2: Overall Survival by response status

Scanned with CamScanner

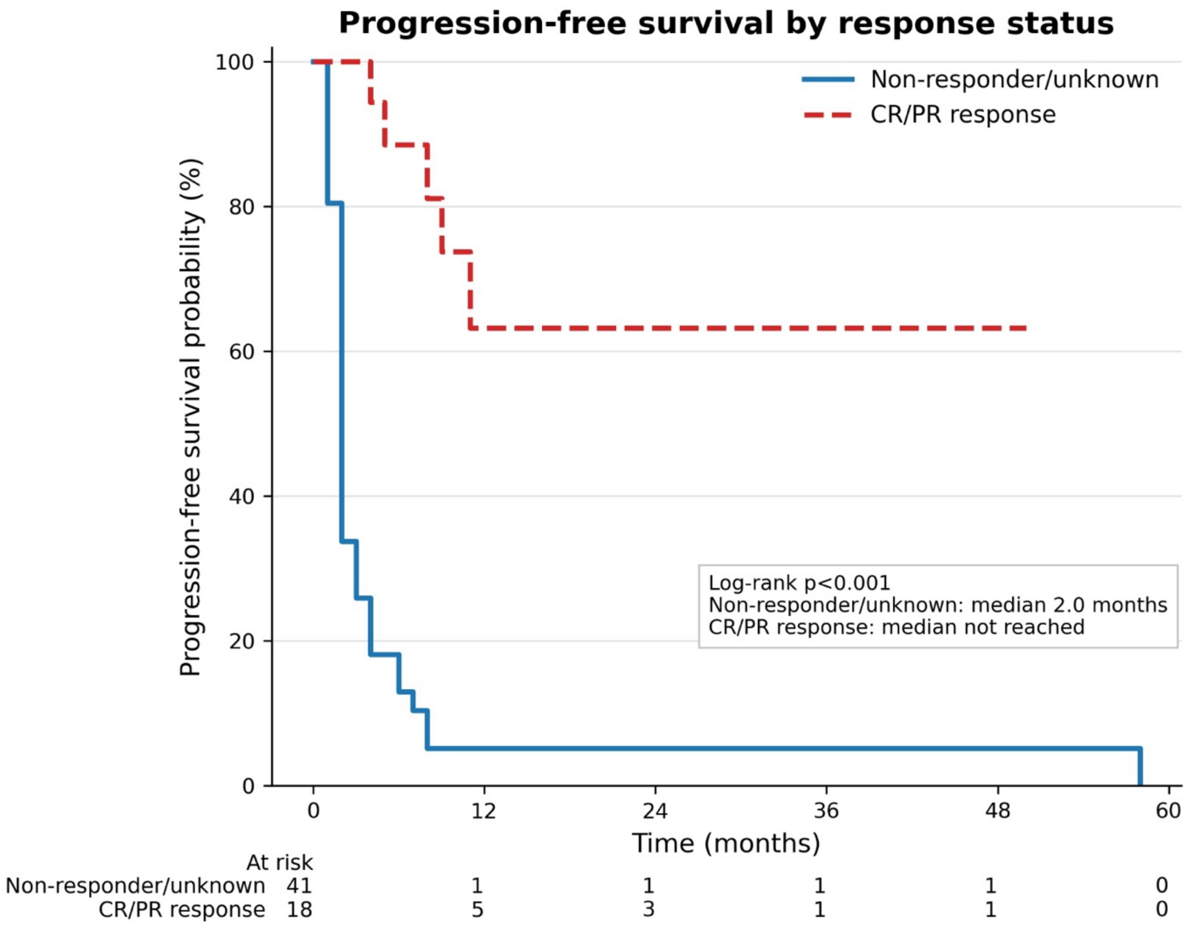

Figure S3: PFS by response status

Scanned with CamScanner
